# First Isolation and Genome Sequence Analysis of *West Nile Virus* in Mosquitoes in Brazil

**DOI:** 10.3390/tropicalmed8040237

**Published:** 2023-04-20

**Authors:** Joaquim Pinto Nunes Neto, Lúcia Aline Moura Reis, Maria Nazaré Oliveira Freitas, Bruna Laís Sena do Nascimento, Liliane Leal das Chagas, Hernan Hermes Monteiro da Costa, Jéssica Cecília Pinheiro Rodrigues, Camila Margalho Braga, Eliana Vieira Pinto da Silva, Sandro Patroca Silva, Lívia Caricio Martins

**Affiliations:** 1 Arbovirology and Hemorrhagic Fevers Section, Evandro Chagas Institute, Ananindeua 67030-000, PA, Brazil; 2 Graduate Program in Parasitary Biology in the Amazon, Center of Biological and Health Sciences, State University of Pará, Belém 66095-663, PA, Brazil; 3 Immunology Center, Adolfo Lutz Institute, São Paulo 01246-000, SP, Brazil; 4 Laboratory Animal Husbandry and Production Section, Evandro Chagas Institute, Ananindeua 67030-000, PA, Brazil

**Keywords:** *West Nile virus* infection, *Culicidae*, *Culex*, disease vectors

## Abstract

*West Nile virus* is a flavivirus transmitted by mosquitoes, mainly of the genus *Culex*. In Brazil, serological studies have already indicated the circulation of the virus since 2003, with the first human case detected in 2014. The objective of the present paper is to report the first isolation of WNV in a Culex (Melanoconion) mosquito. Arthropods were collected by protected human attraction and CDC light bait, and taxonomically identified and analyzed by viral isolation, complement fixation and genomic sequencing tests. WNV was isolated from samples of Culex (Melanoconion) mosquitoes, and the sequencing analysis demonstrated that the isolated strain belonged to lineage 1a. The finding of the present study presents the first evidence of the isolation and genome sequencing of WNV in arthropods in Brazil.

## 1. Introduction

*West Nile virus* (WNV) is a mosquito-borne flavivirus of the family *Flaviviridae*, genus *Flavivirus*, transmitted by the bite of mosquitoes, primarily of the genus *Culex*. Natural hosts are some wild bird species, which act as amplifiers and infection sources for mosquitoes [1,2,3].

WNV is an RNA virus consisting of approximately 11,029 nucleotides (nt), surrounded by a 35-nm inner capsid consisting of multiple copies of a capsid protein (C), and surrounded by an outer layer containing structural envelope (E) and membrane (M) proteins. The genome also encodes seven non-structural (NS) proteins that are involved in the replication and maturation of the virus (NS1, NS2a, NS2b, NS3, NS4a, NS4b, and NS5) [4,5,6].

WNV infection may cause oligosymptomatic to severe and fatal cases of encephalitis [7,8], with 70% to 80% of infected persons presenting asymptomatic cases; 20% to 30% presenting fever, headache, myalgia, malaise, chills, and diaphoresis; and only 1 in 150 infected persons presenting central nervous system (CNS) involvement, with a higher risk of occurrence in patients over 50 years of age and in immunocompromised persons [8,9,10,11,12].

WNV was introduced in the Americas in 1999 in the United States, followed by Canada [13], Mexico [14,15], and Venezuela [16].

In Brazil, serological findings in birds, horses, and other animals have indicated the circulation of the virus since 2003. However, the first human case of West Nile fever was only documented in 2014, in Piauí, through a strategy of investigation of neurological syndromes [17]. In April 2018, a fragment of the viral genome was detected in Espírito Santo due to the occurrence of an equine epizootic with neurological manifestations, and genetic sequencing confirmed the presence of WNV [18]. Recently, in September 2019, the WNV genome was detected in a horse in the municipality of Boa Viagem during equine epizootic investigations in several municipalities of Ceará State [19].

In 2021, the Epidemiological Bulletin (vol. 52, no. 41) of the Secretariat of Health Surveillance highlighted West Nile fever as one of the zoonotic diseases that present a high risk to public health in Brazil [20].

*Culex* mosquitoes, which are considered the primary vectors of WNV, have a cosmopolitan distribution and include approximately 768 species divided into 26 subgenera [21,22]. Many of these species are well adapted to the urban environment, where they find suitable breeding grounds for reproduction, as the females lay their eggs in small collections of stagnant water containing high levels of organic matter [23,24,25].

Arthropod-borne viruses (arboviruses) are a major health threat in several countries, especially those with tropical climates. This is due to the great potential of vectors to spread and adapt to different environments and hosts, and to the fact that the temperature is favorable for viral replication. Climate change and anthropic activities such as deforestation due to uncontrolled occupation, mining, and population migration are also factors that contribute to viral amplification and transmission, as well as the emergence and re-emergence of arboviruses. These factors alter the natural habitats of the vectors, increasing the risk of human exposure to these viruses [26,27,28,29,30].

The objective of the present paper is to report the first isolation of WNV in a *Culex* (*Melanoconion*) mosquito pool collected during a vector surveillance study in the southeastern part of Pará State, Brazil.

## 2. Materials and Methods

### 2.1. Epidemiological Investigation of Arboviruses

The Arbovirology and Hemorrhagic Fevers Section (SAARB) of the Evandro Chagas Institute (IEC), the National Reference Laboratory for the diagnosis of Arboviruses of the Health Surveillance and Environment Secretariat (SVSA) of the Ministry of Health (MS), conducts surveillance and monitoring of arbovirus and other vertebrate virus circulations in different environments, especially in the Northern Region of the country.

Since 2005, IEC has been developing studies in the southeastern region of Pará, covering the municipalities of Marabá, Parauapebas, Curionopolis, and Canaã dos Carajás. These studies aim to assess the impact of mining activities on the emergence and re-emergence of arbovirosis and other endemic tropical diseases in the region, especially those with vectorial transmission.

**Figure 1:**
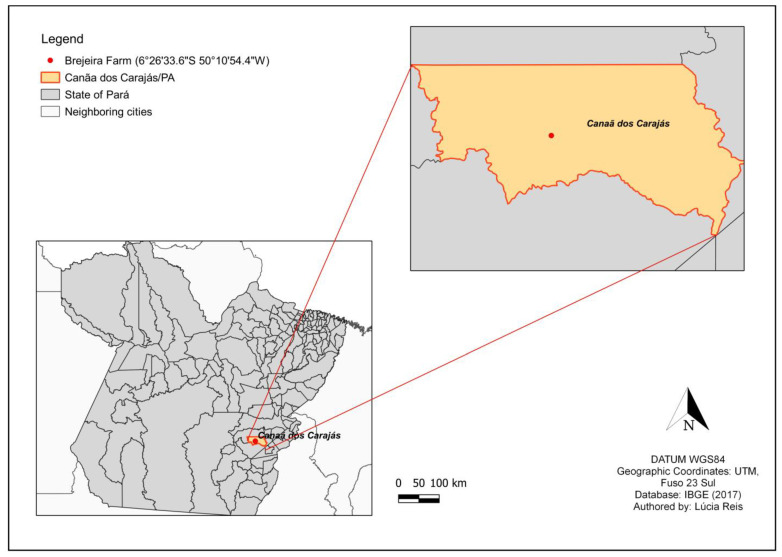
Location of the municipality of Canaã dos Carajás (outlined in orange) and the arthropod collection site (red dot).

In this context, WNV was isolated for the first time in the southeastern region of Pará. It was isolated from a pool of 40 mosquitoes of the genus *Culex* (*Mel*.). The specimens were collected in March 2017 in the Municipality of Canaã dos Carajás/PA (S6°26′33.61″/W50°10′54.35″) (Figure 1). The RT-qPCR technique for WNV confirmed the arthropod infection, followed by genomic sequencing of the virus using the IIIumina platform.

### 2.2. Capture and Identification of Hematophagous Arthropods

Two methods were used to collect hematophagous arthropods, the human-protected attraction technique [31] and the CDC light trap, both in the canopy and on the ground.

The technique of human attraction consists of collecting the mosquitoes that approach the professional, using equipment such as the puçá and the oral suction catcher, before the mosquito begins to hemophagy [31]. CDC light traps consist of an automatic trap that uses light to attract arthropods, and a fan that sucks them into a collection cup attached to the unit [32,33,34,35,36].

After collection, arthropods were placed in cryotubes, stored in liquid nitrogen (~196 °C), and transported to the SAARB Medical Entomology Laboratory (IEC/PA), where they were transferred to a −70 °C freezer. Taxonomic identification was performed.

### 2.3. Viral Isolation in Cell Culture and Indirect Immunofluorescence Test (IIF)

For virus isolation assays, 1000µL of Dulbecco’s buffered saline solution (DPBS) (Life Technologies, Carlsbad, CA, USA) containing 2% penicillin and streptomycin, 1% fungizone, and 5% fetal bovine serum (FBS) was added to the arthropod pools. The samples were macerated in a TissueLyser II equipment (Qiagen, Hilden, Germany) and stored in a −70 °C freezer for a minimum of 24 h, in accordance with the protocol of Vazeille et al. [37].

The samples were inoculated into C6/36 (ATCC: CRL-1660) and VERO (ATCC: CCL-81-VHG™) cell cultures [38], according to the protocol of Igarashi [39]. For inoculation, 100 µL of the supernatant was inoculated into each cell line after thawing and centrifugation (Mikro 220R, Hettich, Föhrenstr, Tuttlingen, Germany). The C6/36 cells were incubated at 28 °C (±2 °C) and the VERO cells were incubated at 37 °C (±2 °C) with 5% CO_2_ for one hour for adsorption. Based on the protocol of Beaty, Calisher, and Shope [40], 1.5 mL of Leibowitz L-15 medium (GIBCO, GRAND ISLAND, NY, USA) was added to the C6/36 cells and 1.5 mL of 199 medium (GIBCO, GRAND ISLAND, NY, USA) to the VERO cells.

To verify the presence of the cytopathogenic effect (CPE), the respective cell line was observed daily under an inverted optical microscope (Olympus CK2 Phase Contrast Microscope, Shibuya-ku, Tokyo, Japan) for seven days. To make the test more reliable, positive and negative controls were used during the process.

The indirect immunofluorescence assay (IFI) using polyclonal antibodies to the genus *Alphavirus*, *Flavivirus,* and *Orthobunyavirus* was performed for serologic identification, according to the protocol of Gubler [41].

### 2.4. Viral Isolation in Swiss Albino Mice

The sample was also analyzed by a viral isolation technique in neonatal mice, based on the protocol established by Rosa et al. [42], in which 0.02 mL of the arthropod macerate supernatant diluted 1:10 in 0.75% bovine albumin in PBS was individually inoculated intracerebrally into six two-to-three day-old Swiss albino infant mice. The animals were observed for signs and symptoms of infection for 21 days. The animals that showed signs of disease were separated and brain and liver samples were collected to identify the infecting agent using the complement fixation test (CF).

### 2.5. Complement Fixation Test (CF)

In order to better characterize and detect the immune response of the viral isolate (viral typing), the complement fixation test was performed. The test was performed by centrifuging the inoculated C6/36 cells for 15 min at 8000 rpm and diluting the pure cell culture 1:2 in Veronal buffer (1:5) according to the protocol developed by Fulton and Dumbell [43] and adapted for plates by Beaty, Calisher, and Shope [40]. Polyclonal sera were then prepared at 1:8, 1:16, and 1:32 dilutions in Veronal (1:5) and inactivated at 60 °C for 20 min. In a 96-well microplate, 25 µL of tested sera, 50 µL of 1:100 diluted complement system (guinea pig serum complement), and 25 µL cell culture suspension were added per well and incubated overnight at 4 °C.

The hemolytic system (developer system) was prepared using equal parts of sheep cells diluted 1:40 in Veronal (1:5) and hemolysin (rabbit anti-sheep cell serum) diluted 1:700 after sensitization in a 37 °C water bath for 20 min. Then, 50 µL of the developer system was added to each well, followed by shaking and incubation at 37 °C. Then, 50 µL of the developer system was added to each well, followed by incubation at 37 °C. Microplates were shaken at intervals of 7, 8, and 15 min, followed by incubation in a refrigerator at 4 °C for at least 4 h.

The results of the test were based on the degree of hemolysis, assigning values from 0 to 4 according to the percentage of hemolysis observed (Table 1).

### 2.6. Molecular Detection and Bioinformatics Analysis

To confirm the isolated flavivirus, RNA extraction was performed using 140 µL of C6/36 cell culture supernatant from sample BeAr848804 for nucleic acid extraction using commercial QlAamp Viral RNA Mini Kit and RT-qPCR for WNV and *Saint Louis encephalitis virus* (SLEV), according to the protocol established by Lanciotti et al. [44], in the ABI7500 Real Time PCR System Equipment (Applied Biosystems) using the Superscript III Platinum One-Step qRT-PCR System Kit (Invitrogen). The choice of both viruses was made because the *Culex* spp. mosquitoes are considered to be the main vectors of both viruses.

The RNA extracted in the preview step was used for the preparation of cDNA started with synthesis of first and second strand cDNA using the SuperScript™ VILO™ MasterMix Kit and NEBNext^®^ Second Strand Synthesis Module, respectively. The PureLink^®^ PCR Purification Kit was used for cDNA purification. All of the steps were performed according to the respective kit manufacturer’s recommendations.

The genomic library was prepared according to the Nextera XT DNA Library Preparation Kit guidelines and sequenced on the Miniseq platform (Illumina, Inc., San Diego, CA, USA) using the MiniSeq High Output Kit v2.5 (300 cycles) using the paired-end method according to the manufacturer’s recommendations at the IEC SAARB, MS/ SVSA, Brazil.

Raw data were processed using Trim Galore v.0.4.5 to remove short reads (<50 nt), adapters, and undetermined bases (reads with more than 15 of N) (https://www.bioinformatics.babraham.ac.uk/projects/trim_galore/ (accessed on 19 January 2021). The reads were assembled using the De Novo Assembly method in IDBA-UD v.1.1 [45], and SPAdes v.3.12.0 [46] to obtain contigs, and the generated data were curated in *Geneious* 9.1.6 (Biomatters Inc., Auckland, New Zealand).

Genomic annotation of the assemblies was performed using the program DIAMOND [47] together with the non-redundant protein database available from the National Center for Biotechnology Information (NCBI), considering the e-value (0.0001) and amino acid identity values.

Phylogenetic inference was performed from WNV strain nucleotide sequences available in the NCBI database using polyprotein coding regions.

Multiple sequence alignment (MSA) was performed using Mafft v.7 [48]. The jModelTest program was used to select the best nucleotide substitution model before performing the phylogenetic analysis [49]. The maximum likelihood (ML) method [50] was used to reconstruct the phylogenetic tree, implemented in RaxML v.8.2.4 [51]. To determine the reliability of the tree topology, bootstrap analysis [52] was performed with 1000 replicates. Phylogeny visualization was performed in FigTree v.1.4.4 (https://github.com/rambaut/figtree/releases/tag/v1.4.4 (accessed on 19 January 2021) and edited in Inkscape v.1.1 (https://inkscape.org/release/inkscape-1.1/ (accessed on 19 January 2021).

## 3. Results

In the study area, located in the municipality of Canaã dos Carajás/PA, a total of 4615 arthropods were collected. Of these, 422 (9.14%) were *Cetaropogonidae*, 1035 (22.43%) were *Psychodidae,* and 3158 (68.43%) were *Culicidae* (Table 2).

**Figure 2:**
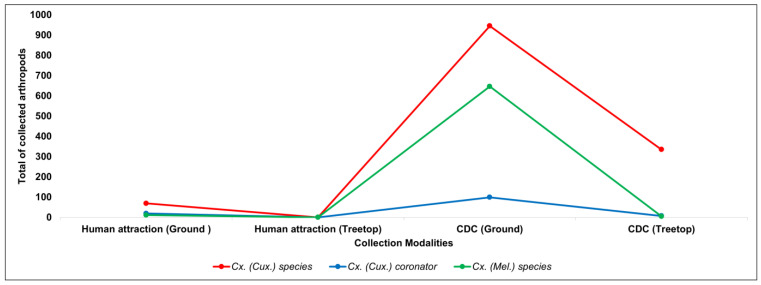
Distribution of the total number of *Culex* arthropods collected, according to the type of collection.

For the genus *Culex* spp. from which WNV was isolated, 2136 (67.64%) mosquitoes belonging to the genus were collected. However, only 125 specimens were identified in terms of species, being *Culex* (*Cux*) *coronator*, and the others were identified only to the genus. CDC in the ground was the collection method that captured a greater number of specimens (Figure 2).

**Figure 3:**
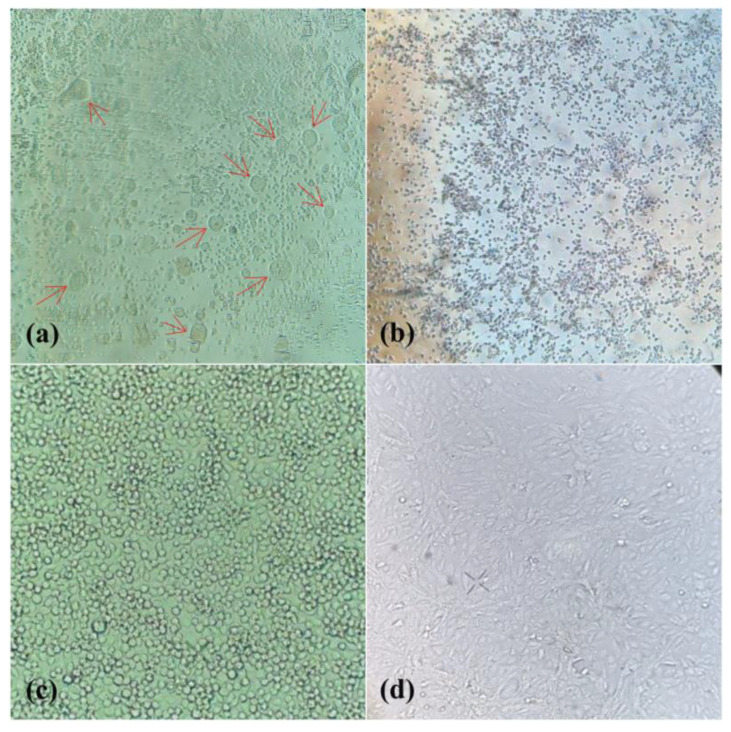
Cytopathic effect (ECP) displayed by C6/36 and Vero cell lines inoculated with sample BeAr848894. (**a**) ECP in C6/36 cells showing formation of syncytium highlighted by red arrows. (**b**) ECP in Vero cells, with modification in the cell morphology and destruction of the monolayer. (**c**) negative control C6/36 cells, showing intact monolayer and normal cell morphology. (**d**) Negative control Vero cells, showing intact monolayer and normal cell morphology.

**Figure 4:**
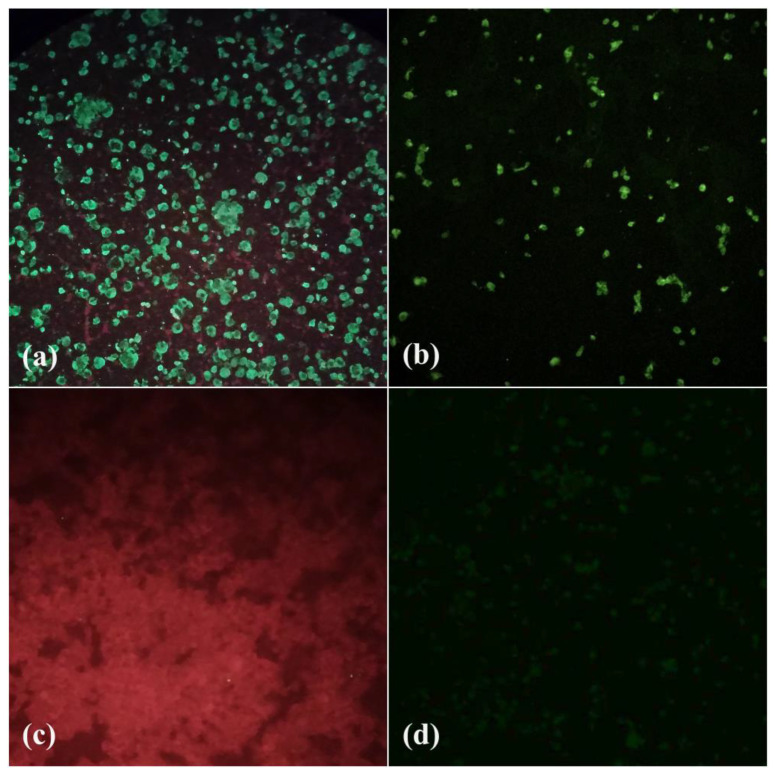
Indirect immunofluorescence reaction (IFI) using polyclonal antibody to Flavivirus. (**a**) C6/36 cells with positive fluorescence reaction. (**b**) Vero cells with a positive fluorescence reaction. (**c**) Negative control C6/36 cells, with cells demarcated using Evans blue staining. (**d**) Negative control Vero cells, showing no fluorescence reaction.

In an arthropod pool (BeAr848804) of *Culex* (*Mel*.) mosquitoes, CPE was observed in the VERO and C6/36 cell lines. Cellular changes in C6/36 cells began at 4 dpi with the formation of syncytia, i.e., multinucleated giant cells, and disruption of the monolayer. Vero cells showed changes in their monolayer from 5 dpi, with changes in cell morphology and disruption of the monolayer (Figure 3). The IFI test showed reactivity with polyclonal antibodies to the genus *Flavivirus* (group B) in both of the cell lines used (Figure 4).

In viral isolation assays in mice, clinical signs of infection were observed from day 11 post infection (dpi), and to increase viral titers, a passage in new mice was carried out in which animals showed clinical signs from day 4 dpi.

In CF, the sample was positive for polyclonal antigen group B antibodies with serologic cross-referencing to WNV and *Rocio virus* (ROCV) (Table 3). This sample was positive for WNV with a Ct of 9.69 in the RT-qPCR test for WNV and negative to SLEV.

**Figure 5:**
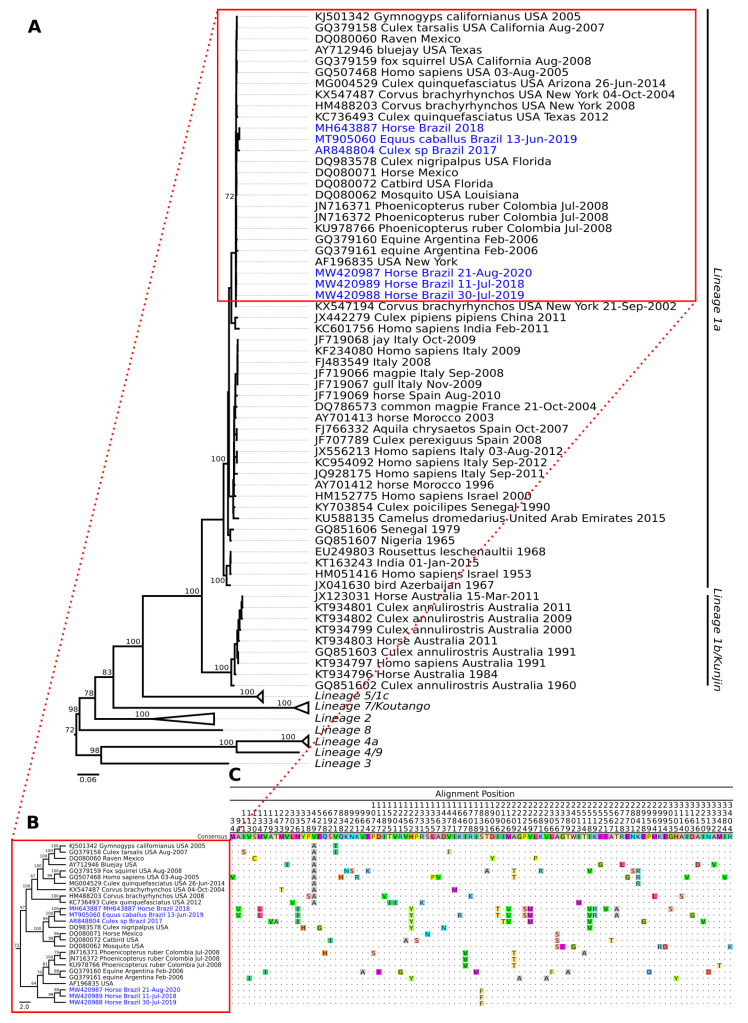
Phylogenetic tree of different WNV strains, including an isolate obtained from mosquitoes of the genus *Culex* spp. in Brazil. (**A**) Using the maximum likelihood (ML) method based on the complete nucleotide sequences of the polyprotein and using the GTR matrix as the best model for nucleotide substitution. Phylogenetic lineages are labeled in different clade colors. The numbers at each main node of the tree correspond to the bootstrap values in percent (1000 replicates). The scale bar corresponds to the nucleotide divergence per site between sequences. Highlighted in blue are the strains identified in Brazil. (**B**) Monophyletic clade of different strains 1a including those from Brazil. (**C**) Aminoacidic alignment of the polyprotein including only the non-synonymous mutations.

The genome of the *West Nile virus* was sequenced, and the complete open reading frame (ORF) of the WNV strain (identified by sequence ID OP422646) was successfully recovered (Supplementary Materials). Phylogenetic analysis showed that this new isolate, similar to the other WNV strains already identified in Brazil, was also grouped in the clade belonging to lineage 1a and was closer to other WNV isolates detected in horses in the State of Espírito Santo in 2018 and 2019 within the same monophyletic clade (Figure 5A), as well as to other strains detected in the US, Mexico, Colombia, and Argentina. However, paraphilia was observed with other samples from Brazil. These were isolated from horses in the states of Piauí, Minas Gerais, and São Paulo (Figure 5B).

Comparison of the nonsynonymous mutations among the isolates from Brazil identified residues (M336I, H1262Y, M2166V, V2259M, and M2518V) that were common only to samples BeAr848804, MH643887, and MT905060 strains, whereas samples MW420987, MW420988, and MW420989 shared a single mutation at position S1839F (Figure 5C).

## 4. Discussion

In Brazil, until 2013, WNV circulation was only detected by serological methods in the samples from several species, including horses and domestic chickens, and was first detected in humans in 2014, in Piauí State. However, the first isolation of WNV occurred in 2018, from an adult horse sample from Espírito Santo State [18,53,54,55,56].

West Nile surveillance in Brazil consists of continuous monitoring of wild and domestic birds and horses with clinical signs of neurological disease or unexplained death, and arthropod surveillance to identify potential vectors of the virus in the country [20]. From this surveillance, new cases of WNV-positive horses with signs of neurological disease in the states of Espírito Santo, Ceará, and São Paulo were identified through genomic studies conducted in 2019 [19,57,58]. Therefore, animal surveillance actions that can participate in the virus transmission cycle are extremely important, as they contribute to the detection of virus circulation in these animals and help to define the areas and populations that are at risk, thus aiming for prevention and control actions.

This paper reports the first isolation of WNV in an arthropod in Brazil, which is directly related to the transmission cycle of this arbovirus in the country. In the analysis of the behavior of the isolate in C6/36 cells, we observed the formation of syncytia by the fusion of plasma membranes of adjacent cells, resulting in the sharing of luminal contents and the formation of multinuclear cells, this behavior is characteristic of enveloped viruses such as WNV [59]. Enveloped viruses are characterized by the expression of proteins that mediate the fusion of the viral envelope with the membrane of the target cells. These proteins are called fusogenic proteins. These proteins are synthesized during the viral replication cycle and are found in the viral envelope and in the cell membranes of infected cells. They adhere to new viral particles during budding. The presence of such proteins promotes cell− fusion, resulting in syncytia formation [60,61,62].

Despite the geographical distance between the locations where the strains were isolated, the isolated strain BeAr848804 is phylogenetically close to the two equine strains from Espírito Santo isolated in 2018 and 2019, forming a monophyletic clade with each other and sharing amino acid mutations, and is part of lineage 1a of WNV, as are the other strains already sequenced in the country. These three strains line up far from three other strains that also circulate in Brazil, in the states of Piauí, Minas Gerais, and São Paulo, forming a divergent paraphyletic clado. The isolate BeAr848804 also aligned closely to the WNV strain isolated in *Culex nigripalpus* (GenBank: DQ983578) in the state of Florida (USA), showing 99% bootstrap value reliability.

Lineage 1 is subdivided into three clades (1a, 1b, and 1c). Clade 1a is found mainly in European, African, Middle Eastern, and American countries. Clade 1b is found in Australia and clade 1c in India [63,64,65].

WNV is maintained in nature in a transmission cycle involving mainly *Culex* mosquitoes [66]. WNV has been detected in 27 mosquito species in the United States, including *Aedes*, *Anopheles*, *Coquillettidia*, *Deinocerites*, *Mansonia*, *Orthopodomyia*, *Uranotaenia*, *Culiseta*, and *Psorophora*, and 14 species of *Culex*, including *Culex quinquefasciatus*, according to the Centers of Disease Control and Prevention (CDC) [67].

Ciota [68] reported that the main WNV transmission cycle is an enzootic cycle between mosquitoes of the genus *Culex* spp. acting as vectors and birds as amplifying hosts in field and experimental studies conducted in Egypt since 1950. It is important to note that the potential of these mosquitoes to transmit WNV, which qualifies them as a vector, is directly related to two factors, vector competence and vector capacity, both of which refer to the feeding behavior and ability of the mosquito to transmit the virus by hematophagy.

In 2020, two pools of *Culex pipiens* mosquitoes in the Netherlands tested positive for WNV RNA. Genomic analysis of the samples showed that the strain circulating in the region belonged to lineage 2, grouping with sequences from Germany, Austria and the Czech Republic [69]. In 2021, WNV was identified in a pool of *Culex pipiens* complex mosquitoes that had been collected in Rosslau, Germany [70].

In North America, *Culex* mosquitoes are the primary vectors of the virus, and patterns of WNV transmission and infection risk are determined by geographic distribution, ecology, and vector capacity [71].

The presence of humans in forested areas, which is considered a terminal host, is another important aspect of the transmission cycle of arboviruses such as WNV. In the region of the Carajás Mineral Complex, various anthropic activities, such as mining, agriculture, and cattle raising, bring people into constant contact with arbovirus vectors, and may also attract them from the forest to urban or peri-urban dwellings. This causes wild vectors to become synanthropic, that is, to adapt to living in areas with humans, which favors the transmission of wild circulation arboviruses in urban areas [72,73].

Thus, environmental changes have great influence on the emergence and proliferation of various diseases, among them, deforestation stands out as the activity that most generates environmental modification, being carried out for the practice of agriculture, timber extraction, road construction, and mining. Such environmental changes also cause disease vectors to change their feeding behavior from predominantly zoophilic to anthropophilic [74,75,76].

## 5. Conclusions

Data from the present study provided the first evidence of isolation and genome sequencing of WNV in *Culex* spp. collected in the municipality of Canaã dos Carajás, Pará, Brazil. This isolate, as well as other isolates previously described in Brazil, was clustered in lineage 1a. In this context, it is important to adopt measures to expand and strengthen entomovirological surveillance as a diagnostic tool for detecting and monitoring arboviruses, as well as identifying other arthropod species that may be vectors of WNV transmission in Brazilian territory.

## Figures and Tables

**Table 1:** Reference values for complement fixation test reading.

Degree of Hemolysis (%)	Reaction Result	Test Value
0%	Positive (+)	4
25%	Positive (+)	3
50%	Negative (−)	2
75%	Negative (−)	1
100%	Negative (−)	0

**Table 2:** Distribution of species collected from human attraction and CDC techniques, at Brejeira Farm, Canãa dos Carajás, March 2017.

Collection Modalities	Ground		Treetop		CDC Ground		CDC Treetop		Total		Percent
Species	Nº	Pools	Nº	Pools	Nº	Pools	Nº	Pools	Nº	Pools	%
*Ae. (Stg.) albopictus*	5	1	0	0	0	0	0	0	5	1	0.16
*Ae. (How.) species*	1	1	0	0	0	0	0	0	1	1	0.03
*Ae. (Och.) species*	7	1	0	0	7	1	0	0	14	2	0.44
*Ae. (Och.) fulvus*	16	1	0	0	19	1	1	1	36	3	1.14
*Ae. (Och.) scapularis*	229	11	0	0	34	1	4	1	267	13	8.45
*Ae. (Och.) serratus*	28	1	0	0	6	1	0	0	34	2	1.08
*An. (Ano.) species*	0	0	0	0	3	1	0	0	3	1	0.1
*An. (Ano.) intermedius*	0	0	0	0	13	1	1	1	14	2	0.44
*An. (Ano.) mediopunctatus*	2	1	0	0	12	1	0	0	14	2	0.44
*An. (Nys.) species*	1	1	0	0	1	1	1	1	3	3	0.1
*An. (Nys.) nuneztovari*	0	0	0	0	1	1	0	0	1	1	0.03
*An. (Nys.) triannulatus*	0	0	0	0	1	1	0	0	1	1	0.03
*Cq. (Rhy.) species*	1	1	0	0	3	1	0	0	4	2	0.13
*Cq. (Rhy.) albicosta*	0	0	0	0	1	1	0	0	1	1	0.03
*Cq. (Rhy.) venezuelensis*	0	0	0	0	3	1	0	0	3	1	0.1
*Cx. (Cux.) species*	69	2	0	0	945	26	335	10	1349	38	42.72
*Cx. (Cux.) coronator*	19	1	0	0	99	3	7	1	125	5	3.96
*Cx. (Mel.) species*	11	1	0	0	646	19	5	1	662	21	20.96
*Hg. (Hag.) janthinomys*	1	1	5	1	0	0	0	0	6	2	0.19
*Li. species*	2	1	0	0	0	0	0	0	2	1	0.06
*Li. durhamii*	6	1	0	0	0	0	0	0	6	1	0.19
*Li. flavisetosus*	5	1	0	0	0	0	0	0	5	1	0.16
*Ma. (Man.) species*	0	0	0	0	3	2	0	0	3	2	0.1
*Ma. (Man.) titillans*	8	1	0	0	1	1	10	1	19	3	0.6
*Ps. species*	1	1	0	0	0	0	1	1	2	2	0.06
*Ps. (Gra.) species*	0	0	0	0	7	1	0	0	7	1	0.22
*Ps. (Jan.) species*	130	6	0	0	4	1	0	0	134	7	4.24
*Ps. (Jan.) albipes*	61	2	7	1	9	1	1	1	78	5	2.47
*Ps. (Jan.) ferox*	125	5	1	1	4	1	0	0	130	7	4.12
*Ps. (Jan.) lutzii*	31	1	0	0	7	1	0	0	38	2	1.2
*Ps. (Pso.) species*	0	0	0	0	1	1	0	0	1	1	0.03
*Sa. (Sab.) belisarioi*	1	1	11	1	0	0	0	0	12	2	0.38
*Sa. (Sab.) tarsopus*	0	0	2	1	0	0	0	0	2	1	0.06
*Sa. (Sbo.) chloropterus*	0	0	8	1	0	0	0	0	8	1	0.25
*Sa. (Sbo.) glaucodaemon*	0	0	4	1	0	0	0	0	4	1	0.13
*Ur. (Ura.) calosomata*	0	0	0	0	100	3	0	0	100	3	3.17
*Wy. species*	63	2	1	1	0	0	0	0	64	3	2.03
Total of *Culicidae*	823	46	39	8	1930	73	366	19	3158	146	100%
% *Culicidae*											68.43%
*Ceratopogonidae*	130	1	0	0	205	1	87	1	422	3	
% *Ceratopogonidae*											9.14%
*Flebotominae* ♀	0	0	0	0	394	4	354	3	748	7	
*Flebotominae* ♂	0	0	0	0	147	1	140	1	287	2	
*Psychodidae*	0	0	0	0	541	5	494	4	1035	9	
% *Psychodidae*											22.43%
Grand total	953	47	39	8	2676	79	947	24	4615	158	100%

**Table 3:** Values referring to the degree of hemolysis of sample BeAr848804 (C6/36) in the complement fixation test (CF).

	Flavivírus			WNV			ROCV		
**Diluition**	8	16	32	8	16	32	8	16	32
**Pure**	4	4	3	4	4	2	4	4	4
**1/2**	4	3	1	4	3	2	4	4	4

## Data Availability

Data is contained within the article and Supplementary Material.

## Supplementary Materials

The following supporting information can be downloaded at: https://www.mdpi.com/article/10.3390/tropicalmed8040237/s1.
